# Diabetes and Metabolism Disorders Medicinal Plants: A Glance at the Past and a Look to the Future 2018

**DOI:** 10.1155/2018/5843298

**Published:** 2018-09-30

**Authors:** Hilal Zaid, Akhilesh K. Tamrakar, Mohammed S. Razzaque, Thomas Efferth

**Affiliations:** ^1^Qasemi Research Center, Al-Qasemi Academy, P.O. Box 124, Baqa-El-Gharbia 30100, Israel; ^2^Faculty of Sciences, Arab American University, P.O. Box 240, Jenin, State of Palestine; ^3^CSIR-Central Drug Research Institute, Lucknow 226031, India; ^4^Department of Pathology, Lake Erie College of Osteopathic Medicine, Erie, Pennsylvania, USA; ^5^Johannes Gutenberg University, Mainz, Germany

Diabetes is one of the world's most widespread diseases, characterized by persistent hyperglycemia and related to metabolic disorder, affecting over 327 million people and causing about 300,000 deaths annually [1]. Despite great advances in prevention and therapy, existing treatments for this disorder have serious side effects. On the other hand, in the modern and contemporary world, the incidence of metabolic disorders and diabetes is rising continuously due to modernization in life style and unhealthy dietary habits, imposing an urgent need for new therapies for the management of this deadly disease. Plants used in traditional medicine represent a valuable source in the search for new medicinal compounds. In traditional system of medicine, at least 1200 species of medicinal plants are used for their antidiabetic attributes. However, only a small proportion of such plants have been scientifically validated [2].

Currently, we are witnessing a great progress in evidence-based modern medicine and pharmacology. The characterization of pharmacological and biological effects of herbal-based medicines is becoming more competitive and complex, with the involvement of experts in this research area belonging to different scientific fields, including botany, chemistry, biochemistry, immunology, molecular biology, and bioinformatics. However, despite this great progress in evidence-based modern medicine and pharmacology, traditional medicinal plants continue to be practiced without regulations in many countries [3]. It is crucial hence to keep on studying medicinal plants efficacy including their toxicity in order to prevent their abuse. This special issue provides a comprehensive overview on physiological as well as molecular and biochemical efficacy of medicinal plants and natural active compounds in treating metabolic disorders and diabetes. For this special issue, the editorial office received 23 papers, and after rigorous peer review process 12 papers were accepted for publication. All published articles focused on the theme of this special issue, dealing with biochemical efficacy, molecular mechanism, or clinical efficacy of plant-derived samples for the management of diabetes or associated metabolic disorders.

C. Rugerio-Escalona et al. evaluated the antihyperglycemic potential of the crude and fractional methanolic extracts of* Hamelia patens* Jacq in streptozotocin-induced hyperglycemic rats. They reported that repeated administrations of the crude or fractional methanolic extract for 20 days lowered blood glucose to a normal level. The extracts were also shown to have significant *α*-glucosidase inhibitory activity and protective effects of kidney and liver function parameters. They further subjected the plant extract for quantitative phytochemical and chromatographic analysis and identified five compounds. Out of those, epicatechin and chlorogenic acid had demonstrated antihyperglycemic effect. Considering its antihyperglycemic potential, the authors proposed the extracts of* Hamelia patens *as efficacious source for the treatment of diabetes.

Increased incidences of diabetes are closely associated with increase in obesity and related hyperlipidemia. Currently available synthetic drugs to lower weight or serum lipid in obese patients have been associated with adverse effect progression of drug resistance. Therefore a well-designed clinical trial to establish the efficacy of an intended molecule is very important. In the clinical study by S. Kang et al., the authors evaluated the impact of* Citrus unshiu* peel pellet (CUPP) on obesity and lipid profile. They selected 118 patients, including 88 females with body mass index > 23 and gave them* Citrus unshiu* peel pellet for 4 weeks and observed a significant decrease in BMI in all subjects after CUPP treatment. They also observed a significant decrease in total cholesterol and triglycerides levels. The authors concluded that CUPP could aid in weight control and improve total cholesterol level, and they proposed a large-scale trial to establish the clinical benefits of CUPP.

In another pilot study performed by P. Algenstaedt et al., the authors test the effect of daily consumption of* Morinda citrifolia* (Noni) fruit juice on the physiological status of type 2 diabetic patients. They treated twenty patients with type 2 diabetes with* Morinda citrifolia* fruit juice for eight weeks with regular monitoring of blood glucose levels and other biochemical parameters. They observed that* Morinda citrifolia* fruit juice consumption decreased fasting blood glucose level in diabetic patients but it did not cause hypoglycemia in normoglycemic patients. They also reported decrease in mean HbA1c and CRP values and improvement in insulin excretion and blood cholesterol status in* Morinda citrifolia* fruit juice treated patients. The authors proposed* Morinda citrifolia* fruit juice as a suitable additive to the diet of diabetic patients.

Enhanced rate of morbidity and mortality of diabetics has become a global health problem and puts a huge economic burden on the world population, urging researchers to clarify the pathogenesis of diabetes using advanced techniques. Proteomic technologies are large-scale research tools that can provide abundant data regarding the pattern of protein expression and are widely used to explore the molecular mechanisms underlying the function of complex bioactive mixtures. X. Qiu and colleagues reported a comparative proteomic analysis of two differently extracted* Coptis Chinensis* (CC) in the treatment of type 2 diabetes. They established a rat model of type 2 diabetes, treated it with supercritical-extracted CC and gastric juice extracted CC, and compared various phenotypic and biochemical parameters between the groups. The report showed a significant decrease in triglyceride, total cholesterol, and low density lipoprotein in both CC-treated groups. Additionally, they reported 15 proteins to be differentially regulated in both CC-treated groups. These proteins were found to be associated with glucose metabolism, insulin action, immunity, inflammation, lipid metabolism, oxidation, and antioxidation. From the data authors established that the two differently extracted CC did not show significant differences in terms of their treatment efficacy.

Type 2 diabetes is characterized by insulin resistance in peripheral tissues, including skeletal muscle, liver, and adipose. Skeletal muscle is the major site for glucose utilization and insulin resistance in skeletal muscle is characterized by impaired translocation of insulin sensitive glucose transporter, GLUT4, to cell surface causing decreased rate of glucose uptake. Therefore, interventions with ability to increase GLUT4 translocation to cell surface can be beneficial for the management of insulin resistance. In the* in vitro* study by S. Kadan et al., the authors assessed the chemical composition, cytotoxicity, and antidiabetic activity of* Gundelia tournefortii* extracts. They revealed 39 compounds in the extracts and found both the methanol and hexane extract to be safe up to 250 *μ*g/ml* in vitro*. Effect of extracts on glucose metabolism was assessed by measuring GLUT4 translocation in muscle cells, and it was found that methanol extract is more efficient in GLUT4 translocation enhancement. The authors proposed that* Gundelia tournefortii* exerts antidiabetic activity by enhancing GLUT4 translocation to the cell surface in skeletal muscle.

Inhibition of *α*-glucosidase and *α*-amylase, which digest dietary starch into glucose, has been an established method for controling blood sugar levels. However, currently available drugs under this category have been reported with unpleasant gastrointestinal side effects that frequently result in therapy abandonment. Therefore, it is necessary to continue the search for new alternatives to *α*–glucosidase and pancreatic lipase inhibitors, with milder side effects and which contribute to the treatment of obesity and type 2 diabetes mellitus, in conjunction with current therapies. With the aim of identifying new pancreatic lipase and *α*–glucosidase inhibitors for the management of diabetes, D. Morales et al. reported potential inhibitor for these enzymes from the hydroalcoholic extract of* Ludwigia octovalvis*. The authors reported identifying and characterizing one flavone (isoorientin) with considerable inhibitory effect of pancreatic lipase and two compounds with potential inhibitory effect against *α*–glucosidases (ethyl gallate and gallic acid). These findings might be useful in the development of a potential novel phytomedicine.

In a similar study, R. S. Shenoy et al. investigated the triterpene glycoside (TG) fraction, isolated from the ethanolic extract of* Gymnema sylvestre*, for inhibitory activity against different glucosidases. TG was characterized by being a mixture of triterpene glycosides, gymnemic acid I, IV, VII, and gymnemagenin and exhibited effective inhibition of yeast *α*- glucosidase, sucrase, maltase, and pancreatic *α*-amylase. TG did not exhibit any toxic effects on *β*-cell viability, showed protection against H2O2 induced ROS generation, and stimulated glucose-induced insulin secretion in *β*-cells. The authors proposed TG as a functional food ingredient and a safe nutraceutical candidate for management of diabetes.

Metabolomics is used to evaluate the characteristics and interactions of low molecular weight metabolites under a specific set of conditions. It is a powerful analytical strategy to identify the metabolites* in vivo* and clarify metabolic pathways. Especially for metabolic disease, such as T2DM, metabolomics offers an opportunity to test multiple metabolic markers in large settings. J. Liu et al. reported a metabolomics-based clinical efficacy of Tangzhiqing tablet (TZQ), a Chinese patent medicine, consisting of five herbs,* Paeonia lactiflora, Morus alba, Nelumbo nucifera, Salvia miltiorrhiza, *and* Crataegus pinnatifida, *for type 2 diabetes mellitus with hypertriglyceridemia. In clinical study with type 2 diabetic patients, they observed that TZQ could reduce glycosylated hemoglobin (HbA1c) and fasting insulin, correlated with the baseline level of triglyceride. In metabolomics data they observed a significant difference between patients and healthy volunteers and identified 17 biomarkers. After 12-week treatment with TZQ they found significant decrease in 11 biomarkers and suggested that TZQ could improve the metabolomic abnormalities in the participants.

Obesity is the major risk factor for increasing incidences of diabetes. Therefore, search towards a molecule that can mitigate both hyperglycemia and obesity is of higher priority for the management of metabolic syndrome. R. O. Malematja et al. evaluated the acetone leaf extract of* Senna italic* for its cytotoxic, antiglycation, and lipolytic effect. They also evaluated its effect on glucose uptake and GLUT4 translocation to cell surface and adipogenesis in 3T3-L1 adipocytes. They found that extract had no adverse effect on cell viability. It showed significant antiglycation effect and decreased the expression of various adipokines. They also observed increased rate of glucose uptake associated with enhanced GLUT4 translocation and expression upon extract treatment in combination with insulin, mediated through the PI3K-dependent pathway. In independent study by J. Lee et al., authors investigated the synergistic effect of Bupleuri Radix and Scutellariae Radix, an herbal combination from Korean medicine on obesity model of 3T3-L1 adipocytes. They reported that combination significantly decreased lipid accumulation and expression of the important adipogenic factor PPAR*γ* and C/EBP*α* and their downstream genes in 3T3-L1 adipocytes. Furthermore, the combination was reported to activate AMP-activated protein kinase alpha, thereby regulating energy metabolism.

In the study by C. Tian et al., the authors investigated the effect and mechanism of action of Wushenziye formula, a traditional Chinese medicine, on skeletal muscle insulin resistance in type 2 diabetes. They observed significant improvement in fasting blood glucose, glycosylated serum proteins, glycosylated hemoglobin, insulin, and insulin resistance index in type 2 diabetic Goto-Kakizaki rats. Under* in vitro* condition, they observed increased glucose consumption, upregulated expression of IRS-1, Akt, and GLUT4, and decreased expression of PTP1B in skeletal muscle cells treated with Wushenziye formula. The author demonstrated that Wushenziye formula regulated glucose metabolism through modulating PTP1B/ IRS-1/AKT/GLUT4 signaling and offered Wushenziye formula as a potential candidate for the management of type 2 diabetes.

The study by Q. Liang et al. aimed to understand the molecular mechanisms of Zuo Gui Wan (ZGW), classic formula in traditional Chinese medicine, on impaired glucose tolerance in mouse embryo. They used high glucose loaded two-cell stage mouse embryos as a model and utilized single-cell RNA sequencing technology for transcriptome analysis. They observed that ZGW reversed high glucose-mediated downregulation of genes in the ribosome pathway, leading to prevention of high glucose-mediated embryo cell death. They also observed that ZGW promoted sugar metabolism via tricarboxylic acid cycle through upregulating the genes in the respiratory chain and oxidative phosphorylation. The study revealed the global gene regulation changes of high glucose affecting two-cell stage embryos and also provided insight into the potential molecular mechanisms of ZGW on the IGT.

Overall, in light of this special issue, plant based therapeutics can be a better option for the management of diabetes and related metabolic complications. However, proper scientific validation of efficacy in established experimental models, clinical efficacy, and assessment for the adverse side effects is of utmost important, for their further development.
